# Institutionally Adopted Perioperative Blood Management Program Significantly Decreased the Transfusion Rate of Patients Having Primary Total Hip Replacement Surgery

**DOI:** 10.1155/2021/2235600

**Published:** 2021-09-30

**Authors:** Hargita Dömötör, Ádám L. Varga, Róbert Sződy, Ferenc Tóth, Gábor Nardai

**Affiliations:** ^1^Department of Anesthesiology and Intensive Care, Péterfy Hospital and National Institute of Trauma, Budapest, Hungary; ^2^Department of Traumatology, Péterfy Hospital and National Institute of Trauma, 1081, Fiumei út 17, Budapest, Hungary

## Abstract

Perioperative transfusion in patients undergoing orthopedic surgery increases the number of postoperative complications. Thus, we have introduced an institution-tailored perioperative blood management program (PBM) to decrease the amount of blood transfused in patients going through primary total hip replacement (THR) surgery. We have conducted a before-after observational cohort study in two predetermined observational periods. Demographic and clinical data, ASA scores, laboratory parameters, features of surgical procedure, and anesthesia were registered. Parameters of perioperative fluid administration, transfusion rate, and postoperative complications were also assessed. One hundred patients in the first and 108 patients in the second observational period were enrolled. Eventhough the ratio of posttraumatic THR procedures increased (9% vs. 17%), the PBM protocol has been utilized effectively and a significant decrease in perioperative blood transfusion rate has been observed (61% vs. 21%). The abolishment of routine preoperative LMWH prophylaxis (90% vs. 16%), intraoperative use of tranexamic acid (10% vs. 84%), and the encouraged exploitation of our postoperative observational facility (5% vs. 39%) were abided by our colleagues. Patients still requiring transfusion had lower preoperative hemoglobin levels (129 vs. 147 g/l), scored higher in ASA (ASA III: 46% vs. 19%), and more often presented postoperative hypotension (40% vs. 7%), oliguria (23% vs. 5%), and infections (9% vs. 2%). We conclude that the individualized perioperative blood management protocol was successfully implemented and yielded a lower transfusion rate and better outcomes. Our study suggests that a partial, institution-tailored PBM program may be suitable and beneficial in countries where the modalities of perioperative blood management are limited.

## 1. Introduction

Total hip replacement surgery is one of the most common orthopedic procedures with a considerable risk of bleeding. We have high-quality evidence that excessive transfusion, besides depleting the national blood reserve, increases the number of postoperative complications, hospital length-of-stay [1], and costs [2]; thus, every healthcare provider has a responsibility to take measures to reduce blood transfusion rates. In our institution, a preliminary audit has shown that among patients undergoing THR, the transfusion rate is above 50% (local audit 2012, data not shown). This result validated the creation of a program aiming to reduce blood use in perioperative care. The program incorporated the elements of the internationally approved perioperative blood management (PBM) concept: preoperative anemia screening and management, reduction of perioperative blood loss, postoperative anemia tolerance, and individualized transfusion therapy [3]. However, whether partial implementation of the program results in transfusion benefit is yet to be clarified. A local clinical advisory board was set up to determine which of the possible measures could both be feasible and have merit in our institution's setting.

## 2. Methods

### 2.1. Development of the Institution-Tailored Protocol (Table 1)

Clinical advisory board (senior anesthesiologist—leader, consultant orthopedic surgeons, and critical care specialists) assembled the interventions integrated into the local PBM program, and the following elements were introduced by institutional guidelines and staff education: (a) careful preoperative screening program for anemia (Hgb < 120 g/l). The preoperative anesthesia visits for patients undergoing THR have been scheduled at least 3 weeks prior to surgery whenever possible. Anemic patients were referred for anemia management, and surgeries were delayed if necessary. (b) Individualized vs. routine administration of preoperative LMWH, based on the patient's risk of thromboembolism. (c) Routine administration of 15 mg/kg intravenous bolus tranexamic acid on incision has been incorporated in our THR protocol as it is shown that tranexamic acid can significantly reduce intraoperative blood loss, and if the presence of contraindications is carefully evaluated, complications are rare [4]. (d) Leaving wound drains after major joint replacement surgery is a controversial topic among orthopedic surgeons. Due to the complexity and controversy of the topic, the omission of wound drain use has not been made mandatory, but only encouraged and ultimately entrusted to the surgeon's judgment. (e) Viscoelastometry-based point-of-care, goal-directed hemostasis treatment using rotational thromboelastometry (ROTEM) has been introduced in the management of major/massive surgical bleeding. (f) The practice of restrictive transfusion is one that requires both extensive training of personnel and the development of facilities that allow continuous monitoring of the patients in the early postoperative period. We have extended the postoperative high-dependency unit with the capacity to observe patients undergoing major joint replacement with moderate-to-high cardiovascular or bleeding risk for the critical first 24 hours led by an anesthetist. (g) We have also conducted institution-wide education among doctors and nurses working in surgical wards on the principles and practice of restrictive transfusion policy. Single unit transfusion and clinical reassessment applicable has also become a recommended, although not obligatory, policy. These measures ensure patient safety while allowing practitioners to determine the patient's individual physiologic tolerance of anemia, thus providing a wider range of tolerable hemoglobin levels. (h) To ratify this novel policy, we have established an administrative control on transfusion that requires practitioners to specify the indication (hemodynamic instability, chest pain, dyspnea, and active bleeding) of transfusion on the request form if the patient's hemoglobin level is above 80 g/l.

Perioperative erythropoietin treatment as a modality of anemia management has not been implemented due to its unavailability in Hungary.

The routine use of cell salvage devices has not been incorporated in our policy either. Eventhough they are proven to be efficient in reducing the transfusion ratio, this concerns mostly procedures with the anticipated blood loss of more than 1000 ml [3]; whereas, in our institution, the average intraoperative blood loss during THR is 300–800 ml (local audit 2012, data not shown); thus, we deemed the cost-benefit ratio unfavourable.

The summary of the measures implemented compared to general PBM elements is given in Table 1.

### 2.2. Data Collection

A periinterventional, single-center, before-after study with prospective data collection was approved by local clinical directory board. Patients: adult patients having primary THR surgery in a three-month period of 2013 and 2016. Data were collected from electronic charts of the hospital informatics system, blood bank, and paper-based charts of anesthesia, surgery, and postoperative care until discharge. TXA dose has been predetermined as 15 mg/kg single bolus infusion at the incision. Oliguria and anuria (urine output < 0,5 ml/kg/h) have been recorded in the 6^th^, 12^th^, and 24^th^ hours postoperatively. Perioperative hypotension was defined as systolic blood pressure under 100 Hg mm measured intraoperatively and in the first 24 hours postoperatively. Comparisons were made between the 2013 and 2016 groups regarding demographics, clinical data, type of anesthesia, basic features, and indication of surgical procedure. We have also examined the utilization ratio of PBM measures, the corresponding change in transfusion rate, complications, and hospital length-of-stay. Long term follow-up (3 months) of infection-related complications (surgical and systemic) has only been performed in the after period (2016) using the data of the electronic hospital chart.

### 2.3. Statistical Analysis

Data are given as mean with 95% confidence interval or as a percentage of the total and the amount of blood transfused as a ratio with the number of patients that received transfusion in the denominator. The significance of differences between groups was analyzed by Mann–Whitney, chi-square, and Fischer tests as applicable. A *p* value < 0.05 was considered statistically significant.

## 3. Results

### 3.1. Comparison of the Population of 2013 and 2016

Demographic and clinical data of the population are given in Table 2.

There were no significant differences between demographic data, preexisting clinical features, modality of anesthesia, length, and technique of surgery between 2013 and 2016 as given in Table 2. Eventhough it did not achieve statistical significance, the increase of posttraumatic surgeries (vs. osteoarthritis) is notable.

There has been a significant increase in the utilization of all institutionally approved PBM measures (Table 3). The use of tranexamic acid has become part of the routine unless contraindications are present. Admission ratio to postoperative high-dependency units increased significantly, granting greater safety to patients with higher anesthetic risk. A restrictive hemoglobin trigger has been kept unless an indication of keeping a higher hemoglobin level was present.

A significant difference has been observed in the transfusion ratio of the before and after groups (61% vs. 21%), eventhough there was no significant disparity in fluid administration, postoperative hemoglobin levels, and hospital length-of-stay. In the after group, patients suffered from postoperative hypotension significantly less frequently (12% vs. 35%), but there was no significant difference in the incidence of oligoanuria. The amount of blood received by patients transfused has shown minimal change (Table 4).

### 3.2. Assessment of Clinical Features and Transfusion Requirements in 2016

Clinical data and complications between transfused and not transfused patients in 2016 are given in Table 5.

Between the transfused and not transfused groups, there was no significant difference in age, surgical, and anesthetic technique. Patients that received transfusion had a significantly higher anesthetic risk (ASA III, 20% vs. 50%), and their preoperative hemoglobin levels were significantly lower. They received tranexamic acid significantly less often (64% vs. 91%) and presented hypotension (7% vs. 40%) and oliguria (5% vs. 23%) in the first 24 hours at a significantly higher rate. Infection rates were significantly higher among patients who needed to receive a blood transfusion (Table 5).

## 4. Discussion

Based on the results above, we can declare that our institution-tailored perioperative blood management protocol was successfully implemented and had a significant impact on postoperative transfusion rates and complications. This supports the assumption that it is not required to execute every element of the PBM program to beneficially influence perioperative transfusion needs.

Improving hemostasis in the entire perioperative period and upgrading postoperative care are the most important pillars of success in our opinion. Research data are conflicted regarding preoperative or postoperative administration of prophylactic LMWH concerning perioperative blood loss. Robust studies found no difference in the effectiveness regarding prevention of thromboembolic events between those whose LMWH prophylaxis were initialized preoperatively and those who were started postoperatively if no indication other than the surgery itself was present [5]. Recent studies focusing on the effect of timing of LMWH prophylaxis on bleeding risk were not conclusive [6, 7]. In patients with the indication of hip fracture, immobilization as a major risk factor is present; thus, this portion of patients received preoperative prophylactic LMWH.

Between 2013 and 2016, the listed contraindications of tranexamic acid administration had become more permissive due to novel clinical data on its safety, thus further augmenting its liberal use.

In 2013, viscoelastometry has not been available in our institution. A ROTEM device was introduced in 2014, and from that point, it has become part of the everyday routine as part of the hemostatic diagnostics and management of trauma and surgical patients. The insights and experience gained using viscoelastometry are in themselves great tools in hemostatic management, and whenever the need arises, viscoelastometric measurements are readily available ensuring patient safety as it is proven invaluable across all surgical fields frequently encountering massive blood loss and consequential multiple forms of coagulopathy [8, 9]. As part of our PBM program using ROTEM-guided hemostatic management has become institutionally recommended with the indication of substantial intraoperative bleeding.

We have observed a major decrease in the incidence of postoperative hypotension. We consider this a consequence of better hemostatic practice and not the more appropriate hemodynamic management. The fact that the amount of crystalloid infusions used remained unchanged and vasopressor use was also similar (data not shown) supports this assumption. There was no detectable difference in the incidence of oliguria in the first 24 hours. There has also been a drastic increase in the number of patients spending the postoperative first 24 hours in a high-dependency unit where their fluid balance was carefully evaluated, thus again increasing the reported incidence. Taking this into consideration together with the substantial drop in the incidence of hypotension and the relatively unchanged amount of administered intravenous fluids, we postulate that the data from 2013 underestimates the incidence of oliguria.

Surgical technique was unchanged between 2013 and 2016, and all our institution's surgeons used the anterolateral surgical approach (Watson-Jones). One has to consider that the improvement of the manual skills of our surgical staff and more vigilant surgical hemostasis may have a role in the reduction of our transfusion rates. Whether this is significant is beyond the scope of our study. Postoperative wound drainage insertion is a conflicting issue. The amount of data is vast, and the results are conflicting regarding both long- and short-term complications [10], the effects on wound healing and infection rate. Concerning the topic of postoperative transfusion rates and hospital length-of-stay, evidence suggests that leaving a wound drain can be detrimental [11]. Based on this evidence, we concluded to encourage but not recommend to omit wound drainage. The introduction of this new practice was detectable in results.

The study shows that yet there are no ultimate predictors regarding the need for perioperative transfusion, there are several shared characteristics among patients deemed necessary to be given blood, and the early recognition and evaluation of these characteristics play a crucial part in adequate fluid and blood management. Among these, the most self-explanatory is lower preoperative hemoglobin levels. Eventhough, before elective surgery, anemia is usually successfully managed, it is obvious that patients at the lower end of the normal range concerning the hemoglobin level are at higher risk for postoperative symptomatic anemia. Of course, it also can be taken into consideration that THR surgery is usually performed at a relatively advanced age and at that the age group with a lower preoperative hemoglobin level may frequently be associated with underlying disease or frailty.

It is shown that patients that needed blood transfusion perioperatively received tranexamic acid at a lower ratio. This could have been a consequence of a lapse in the implementation of this modality, and some of the most common diseases such as anamnestic thromboembolic events and/or medical anticoagulation are among the contraindications of the administration of tranexamic acid.

It is a long-standing and widely accepted fact that postoperative hypotension and oliguria are good, easily assessable markers of hypovolemia and can be predictors of adverse outcome [12]. It is important to note the fact that hypotension and oliguria are not always the result of hypovolemia and/or anemia but can also be caused by the many conditions associated with surgical and other iatrogenic insults that result in the impairment of cardiac output such as decompensation due to fluid overload, vasoplegia, and acute kidney injury. Understandably, patients with the worse clinical conditions are more susceptible to such effects. The fact is that in the transfusion group, the incidence of these in the first 24 hours postoperatively is congruent with our earlier observation that these patients were overall in a worse clinical condition.

It is shown that the transfusion group had an elevated postoperative infection rate. That is in accordance with the aforementioned difference in the overall clinical condition of these patients as well. There are compelling data including many surgical fields that perioperative transfusion is a risk factor of postoperative infection [1, 13–15]. It is up to debate whether there is causality established by the immunosuppressive effects of transfusion [16] or it is merely a statistical correlation deriving from the same premise that those requiring transfusion are either in a worse preoperative clinical condition and/or underwent greater surgical stress.

We acknowledge the limitations of this study; this is a single-center study, and as such, it cannot give information regarding the applicability of the process in different institutes. Pillars and parts of the PBM program would be differentially important in other systems. As a before-after study that incorporated multiple interventions, we cannot determine the ratio of the overall efficiency that could be attributed to a single measure. Regarding the overall efficiency, as mentioned above, we cannot determine to which extent the success can be attributed to the PBM measures and to what extent are the changing surgical techniques and skills responsible.

## 5. Conclusion

We conclude that our institution-tailored PBM program was successful and the consequential results have deemed it a worthy endeavor. We have greatly decreased our perioperative transfusion ratio, thus both reducing the risk of transfusion-associated complications and lifting the burden from our National Blood Reserve. We have also conducted a successful training program resulting in a more professional approach to precision medicine. As a result, short-term postoperative complications were significantly decreased. Our results encourage other hospitals to attempt implementing their own institution-tailored PBM protocols even if application in full is not feasible.

## Figures and Tables

**Table 1:** Elements of the general PBM program as integrated into our institutional protocol.

Anemia screening/correction	Preoperative screening	Yes	Surgery, if Hgb > 120 g/l
	Anemia correction, iron supplements	Yes
	Anemia correction, EPO treatment	No	Off label in Hungary

Minimizing blood loss	Less invasive surgical technique	No	Not changed during study period
	Tranexamic acid administration	Yes	Liberal use
	Autologous blood donation	No	Not supported by national blood service
	Hemostasis optimization, preoperative	Yes	Preoperative LMWH only if high risk
	Hemostasis optimization, intra/postoperative	Yes	Viscoelastometry-guided protocol
	Cell salvage techniques	No	Not beneficial
	Normothermia	Yes

Increasing anemia tolerance	Advanced postoperative care	Yes	Postop HDU introduced
Rational hemotherapy	Restrictive transfusion threshold	Yes	Transfusion, if Hgb < 80 g/l

**Table 2:** Demographic and clinical data of the population. Data are shown as mean (CI 95%) and percentage of the total.

	2013 (*n* = 100)	2016 (*n* = 108)	
Age (years)	68 (65–70)	68 (66–69)	ns
Female (%)	70	64	ns
ASA III. (%)	22	21	ns
BMI (kg/m^2^)	28.1 (27.8–28.9)	28 (27.7–29.0)	ns
Preoperative Hgb level (g/l)	138 (135–141)	126 (123–129)	ns
Antiplatelet therapy (%)	16	10	ns
Indication: hip fracture (%)	9	16,6	ns
Neuraxial anesthesia (%)	80	84	ns
Length of surgery (min)	90 (84.9–95.1)	100 (95–105)	ns
Cemented THR (%)	73	74	ns

**Table 3:** Implementation ratio of the elements of the local PBM program.

	2013 (*n* = 100)	2016 (*n* = 108)	
Preoperative LMWH (%)	90	16	*P*< 0.05
Tranexamic acid (i.v.) (%)	10	84	*P* < 0.05
Wound drainage (%)	100	89	*P* < 0.05
Postoperative HDU admission (%)	5	39	*P* < 0.05
Transfusion trigger < 80 g/l (%)	5	86	*P* < 0.05

**Table 4:** Fluid management, transfusion rate, and complications. Data are shown as mean (CI 95%) and percentage of the total.

	2013 (*n* = 100)	2016 (*n* = 108)	
Crystalloids administered, intraoperative (ml)	3421 (3280–3560)	2872 (2720–3030)	ns
Crystalloids administered, postoperative (ml)	1319 (1160–1480)	2113 (1940–2280)	ns
Min. Hgb. level in postoperative 24 hours (g/l)	101 (98.5–104)	104 (101–107)	ns
Transfusion ratio (%)	61	21	*P* < 0.05
Units transfused RBC/number of transfused patients	3	2,8	ns
Postoperative hypotension/24 hours (%)	35	12	*P* < 0.05
Postoperative oligoanuria/24 hours (%)	5	8	ns
Hospital length-of-stay (days)	12	11	ns

**Table 5:** Clinical data and complications between transfused and not transfused patients in 2016. Data are shown as mean (CI 95%) and percentage of the total.

	Transfusion (*n* = 85)	Transfusion+ (*n* = 22)	
Age (years)	68	73	ns
ASA III (%)	20	50	*P* < 0.05
Length of surgery (min)	98 (93–103)	104 (84–119)	ns
Cemented THR (%)	72	84	ns
Indication: hip fracture (%)	14	27	ns
Preoperative Hgb level (g/l)	143 (140–146)	126 (129–133)	*P* < 0.05
Tranexamic acid i.v. (%)	91	64	*P* < 0.05
Neuraxial anesthesia (%)	86	77	ns
Postoperative hypotension/24 hours (%)	7	40	*P* < 0.05
Postoperative oligoanuria/24 hours (%)	5	23	*P* < 0.05
Infection rate^*∗*^ (%)/<3 months	2	9	*P* < 0.001

^*∗*^Surgical and systemic infections.

## Data Availability

The data used to support the findings are available from the corresponding author upon request.
